# The recurrence of geophysical manifestations at the Campi Flegrei caldera

**DOI:** 10.1126/sciadv.adt2067

**Published:** 2025-05-02

**Authors:** Tiziana Vanorio, Davide Geremia, Grazia De Landro, Tianyang Guo

**Affiliations:** ^1^ Department of Earth and Planetary Sciences, Stanford Rock Physics and Geomaterials Laboratory, Stanford University, Stanford, CA 94305, USA.; ^2^ Department of Physics, University of Naples “Federico II”, Naples, Italy.

## Abstract

The Campi Flegrei caldera (CFc), Italy, exhibits distinct unrest patterns, including shallow seismicity following substantial strain accumulation, all within a densely populated area. Previous geophysical studies typically analyzed individual episodes, but by comparing two distinct unrest periods we identified recurring manifestations and *V*_P_/*V*_S_ anomalies linked to a confined reservoir at 2- to 4-kilometer depth. Integrating rock physics experiments under hydrothermal conditions, 24 years of rainfall data, and subsurface hydrodynamics, we found increasing rainfall rates, which indicate reservoir recharge and pressurization. We show that hydrothermal water promotes caprock sealing through the formation of a fibrous microstructure. Our experiments further demonstrate that fluid accumulation rates directly influence deformation rates. Together, these processes drive gradual deformation, natural seismicity, and deepening earthquake foci. Recognizing these recurring patterns is crucial for understanding the caldera’s unrest-driving mechanism, enabling us to offer actionable insights for hazard assessment and engineering solutions, such as intercepting water upstream to prevent drainage toward Pozzuoli.

## Teaser

Campi Flegrei unrest results from a unique geological convergence, with strategies to mitigate pore fluid pressure.

## INTRODUCTION

Episodes of caldera unrest worldwide present major challenges for individuals, scientific institutions, and local authorities, particularly in densely populated regions. Effective risk evaluation and management are challenging due to the complexities of the subsurface environment and the limitations in interpreting data from remote geophysical monitoring, especially from single episodes of unrest. However, continuous monitoring and long-term data series now enable comparisons across different unrest episodes, identifying patterns and anomalies that might not be evident in single, point-in-time snapshots of unrest. Shifting the focus from individual, isolated events to understanding subsurface processes as part of broader, periodic geological patterns allows for a dynamic, time-dependent understanding of subsurface processes. This approach aids in designing novel laboratory experiments to explain field hydrothermal processes, helping to resolve conflicting geophysical findings (*1*). The Campi Flegrei caldera (CFc) in Italy exemplifies this challenge, undergoing cyclical unrest with debated causes. Traditional risk management strategies such as displacing residents (*2*) fall short, causing severe economic and social disruption while failing to protect critical infrastructures (e.g., electric, municipal water, and methane gas lines).

CFc’s unrest features ground deformation, seismic activity, and gas emissions similar to other calderas (*3*). However, CFc is unique in that it experiences substantial uplift, sometimes reaching levels in the range of meters, while seismic energy is not promptly released despite substantial strain accumulation (*2*, *4*). These events, characterized by slow, rhythmic ground movements known as bradyseism (slow movement), were described by Lyell in the 19th century (*5*). Academic discourse has traditionally emphasized melt refill (*6*–*10*) to explain deformation and seismicity, often overlooking CFc’s fundamental nature as a geothermal field with an active hydrodynamic activity (*11*, *12*). The caldera exhibits signs of phreatic explosions—the most recent occurring in 1538 (*13*)—that have resulted in a volcanic field characterized by multiple craters rather than a single cone.

Advanced seismic imaging techniques, using both active and passive sources (*14*–*19*), have elucidated the CFc caldera’s structure down to 7- to 8-km depth. Moreover, 13 wells drilled in the area during the 70–80s provided stratigraphic data of the horizons, geothermal reservoir characterization, and core samples (*20*–*25*). The caldera features a funnel-shaped structure (*14*–*16*) formed by its collapse, consisting of (i) a deep melt zone at 7 to 8 km in the Mesozoic carbonates of the Southern Apennine nappes (*17*) and (ii) two hydrothermal reservoirs: a shallower water-dominated reservoir (*20*, *21*) up to 1 km deep in the Neapolitan Yellow Tuff with high *V*_P_/*V*_S_ ratio (*8*, *15*, *18*, *19*), the ratio of P-wave velocity (*V*_P_) to S-wave velocity (*V*_S_), and a deeper, gas-enriched reservoir below ~2 km within volcanoclastic marine sediments. This latter consists of siltite (clay, marly silt, and sand) mixed with volcanics (tuffites) (*22*–*24*), characterized by low *V*_P_/*V*_S_ ratios (*8*, *15*, *19*) and permeated by superheated water with a dissolved CO_2_ content varying from 1 to 5% (*20*, *21*). A caprock of cemented pyroclastic material (*21*, *25*) from the Campanian ignimbrite eruption (*16*) is situated between ~1 and 2 km and separates the two reservoirs while capping the deeper one. Figure S1 illustrates the stratigraphic formation of the caldera observed in wells SV3 and SV1 (*20*, *21*) and provides a visual of the core samples from SV wells along their P-wave velocity.

Within the deforming area, the caprock marks the onset of velocity reversals below 2 km (fig. S1C) (*15*), indicating a caprock seal overlying the overpressurized volcaniclastic-siltite formation. The overpressure prevents effective stress from increasing as rapidly as it would under normal lithostatic pressure conditions, thereby maintaining relatively high porosity. Vanorio and Kanitpanyacharoen (*25*) linked the ability of this caldera to experience substantial uplift, while accumulating strain energy, to the fiber-reinforced nature of the reservoir’s caprock microstructure, which enhances mechanical response (*26*–*29*). Vanorio and Kanitpanyacharoen (*25*) hypothesize a natural cementation process resulting from a pozzolanic reaction, typical of cement, between volcanic ash and hyperalkaline fluids such as sodium- and calcium-rich brine found in the wells (*20*). While it is known that fiber entanglement influences the mechanical response (*26*–*28*) and hydrothermal conditions can promote fiber formation (*29*), this process has not yet been experimentally verified under conditions and compositions specific to the caldera. Most recently, De Landro *et al.* (*30*) used a dataset of ~10,500 events from the ongoing unrest (2011–2024), along with an initial three-dimensional mapping of earthquake locations from (*31*), and velocity and strain signatures from rock physics experiments under hydrothermal conditions. This enabled De Landro *et al.* (*30*) to define, with unprecedented resolution, key structures of the CFc and the spatiotemporal evolution of seismicity.

The use of rock physics data from deep wellbores in constraining seismic imaging studies addresses the “known unknowns” described by Hill (*1*), which is crucial for overcoming the limitations of geophysical methods not directly measuring rock physics properties in the subsurface. These rock property insights have provided context for modeling CFc’s deformation history over the past 35 years, proposing a correlation between ground deformation and variations in pore pressure and fluid content within the hydrothermal system (*32*). However, the triggers and mechanisms behind the periodic unrest remain unclear.

This study explores the “unknown unknowns” described by Hill (*1*), including overlooked knowledge and perturbations to the geothermal system of CFc. We identified recurring patterns in unrest manifestations, deformation and seismicity, along with *V*_P_/*V*_S_ anomalies, suggesting a common underlying cause that periodically reoccurs in the area, producing similar temporal and spatial patterns and anomalies. This prompted an investigation into their connection to the potential recharge of the confined reservoir over time, considering subsurface hydrodynamics, the sealing of its caprock, and changes in the rate of deformation due to pressure buildup. The plausibility of these hypotheses was tested in the laboratory through site-specific experiments conducted under both burial pressure and hydrothermal conditions.

Specifically, this study addresses three unanswered and interconnected questions that are crucial for understanding the engine of deformation: What is the potential source of fluids that accumulate slowly over time, and what pressure could it generate? What factors might control the rate of deformation in the fluid-accumulating reservoirs as pressure builds up? How does the caprock reseal and develop a fiber-reinforced rheology capable of accumulating strain energy?

Our study begins by establishing temporal patterns in recorded seismicity and deformation, along with spatial correlations in velocity anomalies within the central region of the caldera. We analyzed unrest from two distinct time periods: the ongoing unrest (2011–2024) and the 1982–1984 episode, during which the peak of the crisis prompted the establishment of continuous and permanent monitoring in the area. Our analysis reveals a recurrence of unrest manifestations and *V*_P_/*V*_S_ anomalies within two key structures in the caldera—the confined geothermal reservoir and the caprock, highlighting persistent features tied to local geological characteristics. By integrating 24 years of pluviometry data, caldera hydrodynamics, and site-specific hydrothermal experiments, we demonstrate that the recharging of the geothermal reservoir is consistent with increased pore fluid pressure from the steady influx of rainwater and CO_2_. Because pore pressure buildup requires a closed system, we experimentally demonstrated: (i) the rapid cementation of volcanic material and its fibrous nature, developing through hydrothermal processes; and (ii) how the rate of deformation depends on fluid accumulation rates. As a back-of-envelope calculation, we also estimated the pore fluid pressure resulting from water-steam accumulation within the reservoir, along with CO_2_ from decarbonation reactions at depth.

Data analyses and experiments enable us to conclude that the unrest in the volcanic-geothermal field of Campi Flegrei represents a perfect storm of geology. The caprock’s ability to self-heal due to the properties of hydrothermal water and the slow hydrological recharge of the geothermal reservoir (i.e., rainfall seepage), over time, work in tandem to seal the system and increase pore fluid pressure while reducing effective stress, ultimately triggering seismicity. The rapid isenthalpic upflow of fluid, driven by liquid flashing to steam during rock fracturing, accelerates reservoir depletion and induces dynamic migration of effective stress both downward and laterally. This stress redistribution propagates from the caprock down to the gas-enriched reservoir and its boundaries, triggering seismicity.

This proposed mechanism for driving the CFc unrest offers a comprehensive explanation for this periodically recurring phenomenon, reconciling the geophysical observations in the area, including seismic velocity anomalies, variations in ground deformation rates, seismicity distribution, and subsidence that concludes the unrest episode. We propose an engineering solution that involves intercepting water upstream through withdrawal from existing wells. This approach aims to alleviate both the financial burden on local communities and the hazards associated with phreatic eruptions in an area that has been affected by this phenomenon for millennia.

## RESULTS

### Temporal patterns of unrest manifestations

**Fig. 1.**
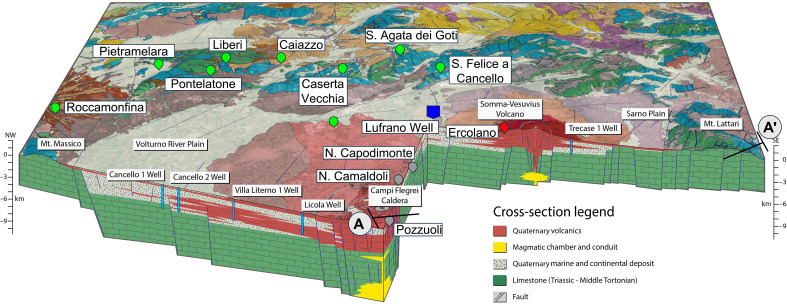
Geological map of the Neapolitan volcanic region, including the CFc and Mt. Vesuvius. Both volcanic systems are situated within the basement of the Southern Apennine nappes’ Mesozoic carbonates and are surrounded by the Apennine chain. Light green symbols represent regional pluviometric stations located on the Apennine Mesozoic carbonates, while gray symbols denote local pluviometric stations near Pozzuoli. Redrawn from the supplemental figure in (*64*); https://creativecommons.org/licenses/by/4.0/. A–A′ indicates the profile of the section shown in Fig. 8. NW, northwest; SE, southeast.

The CFc is a large siliciclastic caldera, collapsed within the Mesozoic carbonate bedrock of the Campanian Plain (Fig. 1) (*13*, *16*). The most recent unrest episodes occurred in 1950–1952, 1970–1972, and 1982–1984, resulting in uplifts of 0.5, 1.7, and 1.8 m, respectively (*4*). Since 2011, a renewed phase of unrest has emerged after ~26 years of subsidence of ~90 cm since 1985, characterized by a period of seismic quiescence. Only the last two unrest episodes have received continuous monitoring, a practice that began amidst the 1982–1984 crisis and led to the establishment of a permanent geophysical network. Geodetic data reveal that uplift is typically concentrated within a roughly 5-km–wide area, peaking consistently within 3-km radius of the central sector of the caldera, where the city of Pozzuoli is located (*2*, *4*). This suggests a relatively small and shallow stress source. The uplift is accompanied by shallow earthquakes and periods of aseismic subsidence.

**Fig. 2.**
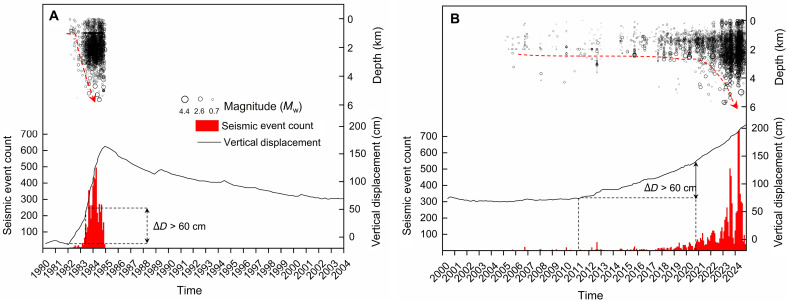
Recurrence of geophysical manifestations at the CFc during two unrest episodes. Panels (**A**) and (**B**) correspond to the 1982–1984 unrest and the ongoing unrest that began in 2011, respectively. Geophysical observations include vertical displacement (black line), seismic events (black circles) represented by magnitude size, and seismic event counts (red bins). Both episodes are marked by substantial vertical uplift of the Earth’s surface and a notable increase in seismic activity as vertical displacement exceeds 60 cm. Seismicity also evolves with depth (red arrow): During the early stages of unrest, seismic events have low magnitudes and are primarily concentrated between 1 and 2 km. As the unrest progresses, earthquake foci deepen, and magnitudes increase. Δ*D*, vertical displacement.

We compared the unrest manifestations from the two most recent episodes, 1982–1984 and the ongoing unrest. Figure 2 illustrates the notable similarities of the manifestations from the two unrest episodes. Both episodes are characterized by (i) notable vertical uplift of the Earth’s surface (black curve in Fig. 2 A and B) and (ii) a notable increase in the rate of seismic events (red bins) when uplift overcomes ~60 to 70 cm, indicating that subsurface rocks can accumulate substantial strain energy before releasing it as seismic activity. Seismicity (black circles) from the peak of the crises is concentrated within the shallow portion of the inner caldera system, with events primarily occurring at depths between 1 km and 4 km, far from the melt region at 8 km (*16*). Both depth and magnitude (circle size) of seismic events evolve over time (Fig. 2). At the early stage of the unrest, seismic events exhibit low magnitudes and are primarily concentrated between 1 and 2 km. As unrest progresses, earthquake foci deepen, releasing greater amounts of energy (*30*–*31*, *33*), shown by the greater magnitude. Data also show a lack of seismic activity between depths of 8 km and 5.5 to 6 km during both unrest episodes. Collectively, these observations suggest that the process initiates at shallow depths, between 1 and 2 km, and gradually migrates downward.

**Fig. 3.**
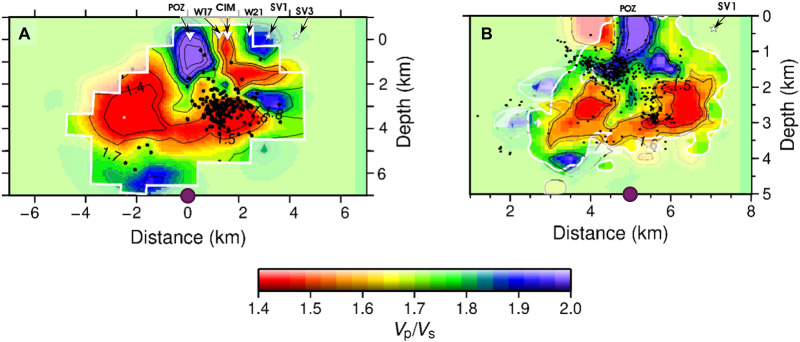
Recurrence of the *V*_P_/*V*_S_ ratio from seismic tomography. (**A**) represents microearthquake travel times during the 1982–1984 unrest (*15*), (**B**) represents the ongoing unrest (*30*). Despite the notable difference in dataset sizes and source-station configuration, resulting in a different volume of investigation and resolution, the recurrence and spatial correlations within the low *V*_P_/*V*_S_ anomaly are notable. The low *V*_P_/*V*_S_ ratio suggests a pressurized, gas-enriched confined reservoir (*15*), as the presence of compressible gas in the pore spaces of rock greatly reduces *V*_P_. The origin (0 km) of the investigated volume in (*15*) corresponds to 5 km in (*30*) (purple dot) and aligns with the location of the city of Pozzuoli.

We also compared seismic imaging based on microearthquake travel times from the 1982–1984 unrest (*15*) and the ongoing unrest (*30*) (Fig. 3, A and B, respectively). Despite the substantial difference in source-station configuration—1209 events for 1982–1984 (*15*) and ~8000 events for the ongoing unrest (*30*), resulting in a different resolution and depth of investigation, the spatial correlation in *V*_P_/*V*_S_ anomalies is notable. Both periods consistently show a low *V*_P_/*V*_S_ ratio between ~2 and 4 km (shades of red), highlighting a recurring feature across the two unrest phases. Rock physics modeling (*15*) and experiments (*30*, *34*) enabled the association of the low *V*_P_/*V*_S_ ratio as indicative of a pressurized, gas-enriched confined reservoir—as steam, CO_2_, or both together. Presence of melt would result in a high *V*_P_/*V*_S_ ratio (*15*) as *V*s would decrease much faster than *V*_P_ (*35*). The comparison of geophysical signatures with wellbore stratigraphy (fig. S1) shows that this low *V*_P_/*V*_S_ horizon corresponds to the confined reservoir hosted within a volcaniclastic siltite (tuffite and quaternary marine and continental deposits) (*20*, *21*), with caprock lying between 1 and 2 km above it. The evolution of seismicity described above, along with the stratigraphy, indicates that seismic events start at shallow depth in the caprock and, subsequently, shift downward to the confined reservoir.

The identification of recurrence in a low*V*_P_/*V*_S_ between 2 and 3 km (Fig. 3), along with the onset of seismicity at shallow depths following a downward migration of seismicity (Fig. 2), suggests a process where effective stress changes dynamically over time, propagating downward from the caprock to the reservoir. The condition for initiating rock failure is expressed in terms of the effective stress, given by

$$\tau_{\text{crit}}=\mu \left(\sigma_{n}-P\right)+\tau_{o}$$ (1)

where the critical shear stress τ_crit_ is the product of the coefficient of friction μ and the difference between the applied normal stress σ_n_ and the pore pressure *P*. This difference is known as the effective normal stress (*36*, *37*). Because the phenomenon is highly localized and tectonic stresses are unlikely to change markedly in this small region, we focused on the variations in effective normal stress due to dynamic changes in pore fluid pressure. Specifically, our analysis addressed the following: (i) identifying the fluid supply source responsible for the observed slow ground deformation in the area, including uplift, its rate, and subsidence; (ii) the recharge of the confined reservoir with low *V*_P_/*V*_S_ characteristics; and (iii) the sealing mechanism of the caprock under hydrothermal conditions, an essential synergy that drives the reduction in effective stress.

### Pluviometry data and caldera hydrodynamics

We examined the hydrodynamics of the CFc, including both potential sources of fluids that could feed the caldera system and the circulation directions of the underground flow. Given the patterns of unrest (*38*) and the hypothesized “breathing” of the caldera (*32*) due to slow deformation, the fluid supply source must accumulate slowly yet able to cyclically trigger seismicity when it reaches a critical threshold of fluid mass for pore pressure buildup. In other words, while a single drop of water has little impact in a depleted reservoir, it can be crucial in a fully saturated one.

**Fig. 4.**
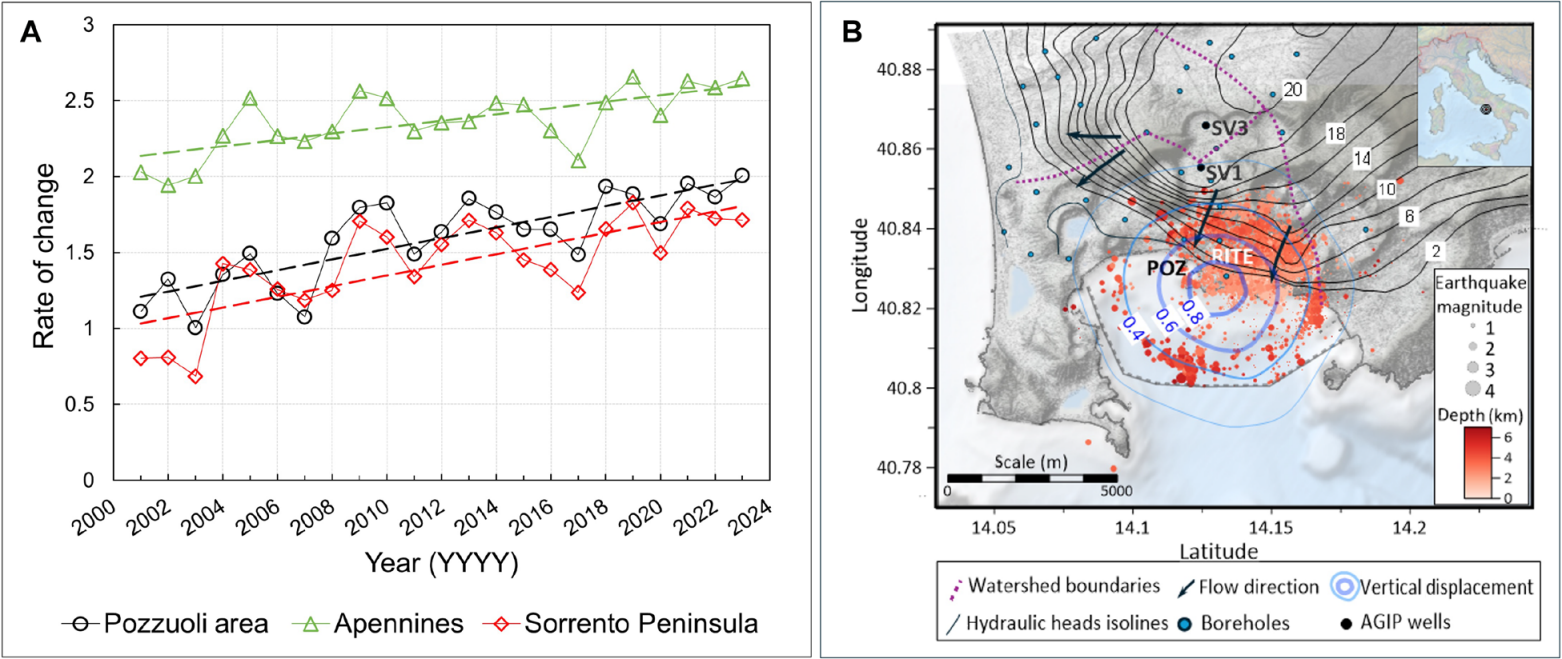
Rainfall data and subsurface hydrodynamics at CFc. (**A**) Average rate of change of rainfall as a function of time, measured at both local stations near Pozzuoli (black) and regional stations surrounding the Campanian plain located north and north-west (green) and southeast of Pozzuoli (red). The locations of pluviometric stations are shown in Fig. 1, with data from individual stations provided in fig. S2 (*39*). (**B**) The epicentral distribution of seismic activity is color-coded by depth (red shades) and overlaid with hydraulic head contours [black isolines, in meters above sea level (a.s.l.)]. The hydraulic head isolines (*40*, *41*) indicate flow radiating outward and draining toward the sea (black arrows), including the town of Pozzuoli, where two ground deformation monitoring stations (POZ and RITE) are located. The watershed boundaries (purple dotted line) define the drainage area encompassing the region experiencing ground deformation. Blue isolines represent vertical deformation, with labels indicating uplift from 2011 to 2024 in meters.

We analyzed the regional and local rainfall data over the past 24 years (*39*). Figure 4A shows the yearly percentage changes of rainfall over time, measured at both local stations near Pozzuoli and regional stations surrounding the Campanian plain (Fig. 1). The regional stations are situated on the Apennine Mesozoic carbonates, which eventually feed the deep-water circulation within the carbonate rocks underneath the caldera. Data from the single stations are reported in the Supplementary Materials (fig. S2). The year-to-year percentage change examines the variations of rainfall over time while addressing the limitations of assessing single points in time. Precipitation does not follow a systematic pattern from year to year, with some years experiencing rainfall higher or lower than the average. In considering each measurement in relation to the previous one, rather than evaluating them individually and independently, the change captures how water volumes evolve over time, thus being a proxy of how cumulative rainfall contributes to water level in reservoirs. Data in Fig. 4 show that year-to-year percentage of rainfall has increased over the past 24 years, which means that the rate of change in rainfall has been growing, not necessarily the total annual rainfall itself. We also examined the underground fluid circulation within the caldera system (*40*, *41*). Figure 4B shows the isolines of the hydraulic heads of the shallower reservoir within the caldera along with the epicentral distribution of seismic activity and the isolines of the deformation. The area experiencing the maximum deformation is centered around the city of Pozzuoli, as indicated by the POZ and RITE stations. Here, our focus is on emphasizing the geometry and direction of the subsurface hydrodynamic flow rather than its actual magnitude, which varies over time. Due to the caldera collapse, the watershed boundaries (purple dotted lines) create a drainage system within the caldera that radiates outward and downward, ultimately collecting downstream toward the town of Pozzuoli, the lowest hydraulic point in the local drainage basin where groundwater converges and accumulates. Figure 4B also shows that the boundaries of the caldera’s catchment area (purple dotted lines) encompass the region affected by ground deformation (blue lines). This indicates that the deformation processes are largely contained within the natural drainage and recharge system of the caldera, suggesting a hydrological and geomechanical connection between the two. This could imply that fluid flow within the catchment area influences ground deformation, possibly due to subsurface fluid accumulation, pressure changes, and hydrothermal activity. Figure S3 illustrates a continuously increasing water head level in a well during the past 24 years and during the 1982–1984 period (*42*). These periods are also associated with a lower pumping rate, suggesting that reduced water usage also contributes to fluid accumulation, as reported by (*42*) for a well in the Neapolitan area.

Additional fluids, both of volcanic origin (*43*) and from thermometamorphic processes (*25*), can further contribute to fluid accumulation and pore fluid pressure buildup, when degassing is impeded. We explored the generation of fluids that results from processes triggered by the gradual accumulation of groundwater within the carbonate basement (*14*, *16*) hosting a melt region (*17*). In high-temperature environments, water circulating through limestone exposed to heat promotes CO_2_ formation through decarbonation (calcination) reactions, leading to the formation of CO_2_ and CaO (lime). While these reactions typically require temperatures around 800°C in dry systems, they can occur at as low as 300°C in the presence of water (*44*)

$$\begin{array}{c} 2\text{Ca}{\text{CO}}_{3\left(\text{calcite}\right)}+{\text{SiO}}_{2\left(\text{quartz}\right)}\\ +H_{2}O↔\text{CaSi}O_{3\left(\text{wollastonite}\right)}+\text{Ca}O_{\left(\text{lime}\right)}+2{\text{CO}}_{2}+H_{2}O \end{array}$$ (2)

The occurrence of decarbonation reactions within the CFc is supported by several observations, including (i) the presence of wollastonite and actinolite in core samples from wells (*20*), indicative of thermometamorphic reactions within the carbonate basement (*25*); (ii) isotopic analysis of CO_2_ from fumaroles showing that about 20 to 40% of the CO_2_ is of sedimentary origin (*45*, *46*); and (iii) the brine composition from wells being rich in calcium oxide [tables 1 to 3 of (*20*, *21*)].

We estimated the pressure generated by CO_2_ formation at a depth of 3 km, where temperatures reach 300° to 400°C, to be ~57 MPa for a complete decarbonation reaction. This estimation hypothesizes that 1 m^3^ of water infiltrates the subsurface, to test the plausibility of the hypothesis (see the Supplementary Materials for details). This CO_2_ pressure adds up to the pressure from other fluids accumulating in the caldera, such as water and vapor (~8.5 MPa at 300°C; see the Supplementary Materials for details). If the reservoir pressure is higher than the saturation pressure at 300°C (8.5 MPa), then water will stay in the liquid state. Vanorio and Kanitpanyacharoen (*25*) reported that the fracture strength of CFc caprock ranges from 45 to 74 MPa depending on depth (i.e., varying confining pressures).

### Hydrothermal water cementation and caprock sealing

Steady accumulation of groundwater and CO_2_, as potential fluid supplies for the geothermal system’s recharge, combined with early-stage seismicity observed at shallow depths within the CFc caprock (Fig. 2), underscores the importance of understanding the caprock’s sealing ability to create a closed system where fluids are trapped, allowing pore pressure to build up. We examined how the reservoir’s caprock can self-seal under hydrothermal conditions, as well as the potential for developing a fiber-growth microstructure (*25*) that contributes to a rheology capable of accumulating substantial strain energy.

**Fig. 5.**
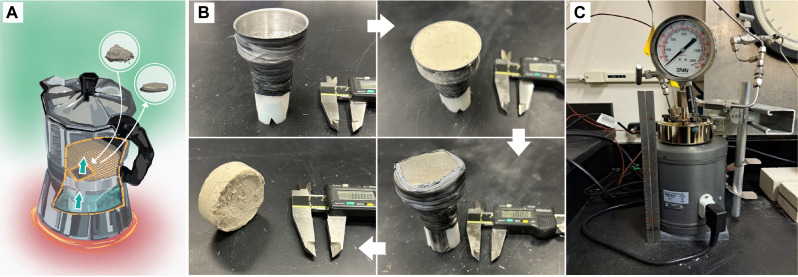
Cementation of volcaniclastic material from ultra-potassic volcanism occurring through a hydrothermal process. The process replicates the rock composition, temperature, and calcium-rich fluids found at depths of 1 to 2 km within the caldera (*20*, *21*), where the seal of the CFc geothermal system is located. (**A**) A conceptual “moka” diagram illustrates the setup: a bottom chamber representing the deeper reservoir, a funnel filter within the hydrothermal vessel (fig. S4) simulating the caprock, and a hot plate mimicking the heat source from the deep melt region (*17*). (**B**) Sample preparation stages, from an empty filter to sample recovery. The funnel filter contains volcanic ash and crushed trachyte-phonolite compositions, simulating fractured caprock after an earthquake cycle, with a conduit allowing vapor to rise. (**C**) Parr reactor, hydrothermal vessel containing the sample holder from (B). Design credit for (A): D. L. Mantle, Stanford University; Software used: Concepts App by TopHatch.

**Fig. 6.**
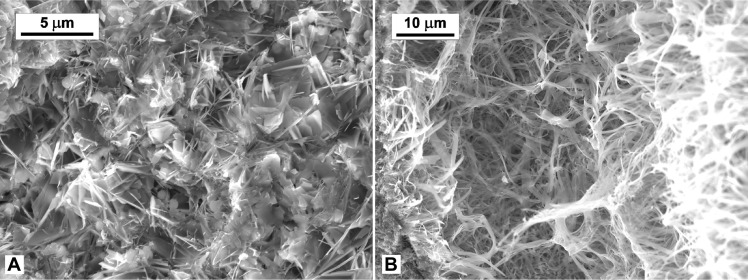
SEM images of the cemented volcaniclastic material from ultra-potassic volcanism through the hydrothermal process. Scanning electron microscopy (SEM) images show the presence of fibrous phases that are entangled and interspersed within the matrix and pore space, reflecting the microstructure of the caldera seal (*25*).

We conducted experiments using rock compositions and pressure-temperature (PT) conditions pertinent to the caldera. Temperature conditions of the experiment replicate those from a well within the caldera rim at 1- to 2-km depth (180° to 250°C, SV1 well). Conducted in a hydrothermal vessel (Fig. 5C) with a core holder that mimics the funnel-shaped structure of the CFc, this setup functions much like a moka pot (Fig. 5A). We designed the experiment with a bottom chamber filled with Ca-rich brine to represent the fluid circulating within the confined reservoir [tables 1 to 3 of (*20*, *21*)], and a funnel filter positioned above the reservoir through which vapor rises via its conduit to promote vapor-transport reactions (Fig. 5B and fig. S4). The funnel filter contains crushed rock to simulate a fractured caprock seal that follows an earthquake cycle (*47*). This material is made up of unconsolidated volcanic ash and crushed trachyte-phonolite rocks from ultrapotassic volcanism typical of the Tyrrhenian margin. The system is then heated at 200°C by a heat source, simulating the magma chamber at depth (*17*). Within just 24 hours of hydrothermal synthesis, the experiment revealed rapid self-healing through cementation (Fig. 5B), which is driven by the hydrothermal water’s properties. Between 150° and 250°C, hydrothermal water acts as a potent solvent, with a high ionic product (*K*_w_) and a dielectric constant like powerful solvents (*48*). SEM analysis of the self-healed material shows the presence of fibrous phases interspersed within the matrix and pore space (Fig. 6A) and entangled (Fig. 6B). This experiment advances our understanding of processes in hydrothermal systems and demonstrates that the hydrothermal conditions present in the caldera can promote rapid self-healing through the formation of mineral fibrous phases, reflecting the fibrous microstructure of the CFc seal (*25*).

## DISCUSSION

### Geophysical observations and patterns of unrest

The CFc experiences periodic unrest, requiring careful hazard assessment and proactive risk management, especially considering the region’s strong anthropization and extensive infrastructure, including methane lines, within an area of high deformation. By analyzing the two most recent unrest episodes, we identified recurring spatial and temporal patterns in the manifestations of unrest and seismic anomalies, including (i) an uplift phase exhibiting variable rates, followed by eventual subsidence; (ii) a seismogenic zone consistently located at shallow depths between 1 and 4 km, with seismic activity migrating downward from 1 to 2 km to 4 km (Fig. 2); (iii) a recurrent low *V*_P_/*V*_S_ anomaly between 2 and 4 km (Fig. 3), consistent with the location of a confined, gas-enriched reservoir at an isotherm of 350°C, where the state of the fluid it contains is pressure dependent, from superheated water at high pressure to vapor dominated as pressure decreases. Despite the difference in resolution, the consistency of this anomaly across two tomographic studies (*15*, *30*) from distinct unrest episodes—each characterized by very different datasets, both in amount of data and source-station configurations—reinforces the reliability of its recurrence; (iv) an increase in seismic event rates when uplift exceeds ~60 to 70 cm, indicating a subsurface rheology that accommodates substantial strain energy before being released as seismic activity (Fig. 2); and (v) the lack of seismicity between depths of 8 km (melt depth) and ~5 km (the deepest yet sparse recorded earthquakes) during the unrest episodes.

If rising magma or magmatic fluids were the primary drivers of the unrest, then we would expect seismicity originating from depths of around 8 km, where melt has been detected in seismic reflection studies (*17*), rising then to 5 km, where the deepest seismicity is recorded. However, Fig. 2 shows that seismicity begins at shallow depths (1 to 2 km) and then it deepens. If melt were intruding from 7 to 8 km and rising to shallower depths, then we would observe the opposite trend. Additionally, the CFc experiences both uplift and subsidence. Following the 1982–1984 unrest, Pozzuoli has undergone ~26 years of sustained subsidence (~90 cm), marked by a period of seismic quiescence. If magma had ascended or hot bodies had intruded during the uplift phase, then it remains unclear where these masses would be displaced to enable the observed subsidence. Once emplaced, dykes and sills occupy both solid mass and volume. For subsidence to occur after uplift, the mass that entered the system must exit, which can plausibly be explained by fluids leaving the system, whether in the form of vapor, gases, or liquid water.

Instead, the observed recurrence in unrest manifestations within the first 4 km suggests that the underlying mechanism has remained consistent over time and is periodically triggered. These observations highlight the importance of understanding the role of the structural features within the first 1 to 4 km of the local geology, including the recharge of the reservoir at depth. Recent studies have reported a link between intense snow fall and stress changes (*49*) as well as rainfall and seismic swarms (*50*–*52*). Compared to previous work, this paper focuses on the year-to-year percentage change in rainfall, rather than the magnitude of rainfall on any single day. This approach emphasizes the growing rate of rainfall (i.e., the additive effect) change over time, which contributes to the rate of recharge of reservoir water levels. It aligns more closely with how ground deformation data is represented. While the ground may uplift or subside daily or weekly by a certain amount, the focus is on the cumulative deformation over time, exerted by a continuously loading source. The importance of this approach is twofold. First, relying on the magnitude of rainfall on any single day may not be entirely pertinent, as its effect on pore fluid pressure and effective stress depends on the reservoir’s saturation state (or recharge) at that specific moment, essentially, the critical threshold for pore fluid pressure buildup mentioned above. Second, in a scenario of poroelastic deformation, where strain and changes in volume related to fluid mass content (ζ) are linked to stress (σ) and pore pressure (*P*) through the rock’s elastic constants (*K* and μ) (see equations below), the volumetric change (with the coefficient 1/*H* controlling the bulk volume change induced by a pore pressure change) is directly influenced by the fluid mass entering the system. *R* represents the change in fluid content within a porous medium due to a change in pore pressure. Therefore, the change in the rate of deformation is expected to closely relate to the rate at which fluid mass enters the system, essentially the reservoir’s recharge rate

$$\varepsilon_{\mathrm{ij}}=\frac{\sigma_{\mathrm{ij}}-\sigma_{m}\delta_{\mathrm{ij}}}{2G}+\frac{\sigma_{m}\delta_{\mathrm{ij}}}{3K}-\frac{p\delta_{\mathrm{ij}}}{3H};\zeta =\frac{\sigma_{m}}{H}-\frac{P}{R}$$ (3)

**Fig. 7.**
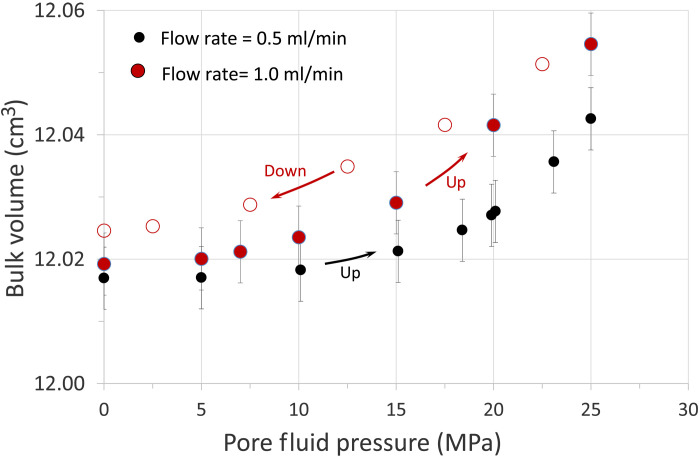
Laboratory experiments showing sample bulk volume change as a function of pore fluid pressure. The experiments are conducted on Berea sandstone where the applied confining pressure (25 MPa) is kept constant to simulate burial pressure at a specific depth. Fluid is introduced into the rock, simulating a closed system where pore pressure lines are sealed. This process increases pore fluid pressure, leading to sample inflation under constant confining pressure. Sample bulk volume rises exponentially as pore fluid pressure increases (up cycles), consistent with findings reported in (*30*). The rate of change in bulk volume depends on the rate at which water is introduced into the rock, with a higher rate of water influx resulting in a greater rate of volume change (solid red cycles). The magnitude of the bulk volume change is directly influenced by the mass of fluid entering the system: The greater the fluid mass and the pressure, the greater the magnitude of inflation. The experiment also shows that sample deflation occurs when pore fluid pressure is released, allowing the fluid to exit the system (red open circles). Sample deformation does not fully recover, showing hysteretic behavior.

Figure 7 presents an example of this correlation. It refers to laboratory experiments conducted on slightly shaley sandstones (Berea) where confining pressure is kept constant to simulate the burial pressure at a specific depth. Fluid is introduced into the rock, simulating a closed system (i.e., pore pressure lines are sealed), which increases the pore fluid pressure causing the bulk volume of the sample to increase (inflation). The plot in Fig. 7 illustrates the change in bulk volume, determined by measuring variations in the sample’s length and diameter as a function of pore fluid pressure and demonstrates that higher fluid flow rates (increased water influx) lead to a faster rate of change in bulk volume (sample deformation). Key observations include (i) bulk volume increases exponentially with rising pore fluid pressure during up cycles, consistent with findings in (*30*) from well cores of the volcaniclastic siltite in the confined reservoir at CFc; (ii) notably, the rate of bulk volume change depends on the rate of water influx into the rock, with higher water influx leading to a greater volume change rate (the slope of the solid red cycles). This experiment also demonstrates that the total change in the sample bulk volume is directly controlled by the cumulative volume of fluid entering (or forming within, in the case of reactions) the system: The greater the pressure, increasing cumulatively with fluid influx, the larger the magnitude of inflation. The experiment also shows that sample volume deflation occurs when pore fluid pressure is released, allowing the fluid to leave the system (red open circles). After being deformed, the sample is not perfectly elastic over the 24-hour duration of the experiment, as the strain is not fully recovered, displaying hysteretic behavior.

### Proposed mechanism and fluid-dynamic processes

**Fig. 8.**
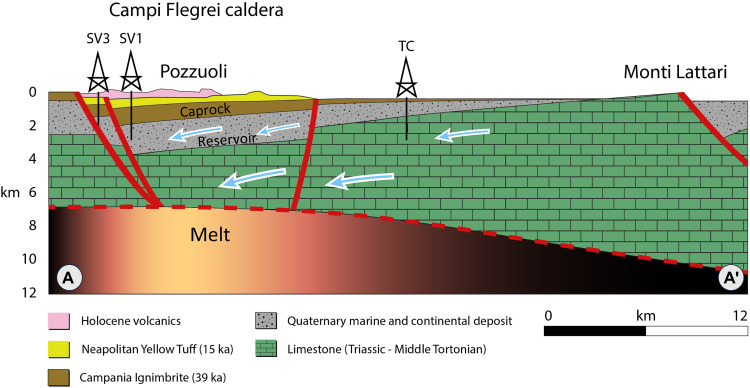
A regional view of the funnel-shaped CFc structure derived from deep seismic reflection data and transmission tomography. The section highlights the CFc collapse within the Mesozoic carbonates of the Southern Apennine nappes, which dip beneath the caldera. The section also highlights the top of the melt zone (*17*), wells drilled in the area (*20*, *21*), faults defining the caldera’s boundary (*14*, *65*), and the primary lithologies present. The structure’s geometry, together with the subsurface flow illustrated in Fig. 4B, outlines a catchment area where Pozzuoli acts as the ultimate drainage point, where water converges. The position of the SV wells is projected on the section. The section is adapted from (*16*) and incorporates feature depths derived from transmission tomography (*14*). SV, San Vito; TC, Tre Case; ka, thousand years (ago).

Regarding the periodic unrest at CFc, no specific mechanisms have been proposed, nor their functioning has been explained in correlation with the geophysical observations. The proposed mechanism leverages the geological structure of the caldera (Fig. 8): A funnel-shaped structure collapsed within the Mesozoic carbonates of the Southern Apennine nappes (*14*, *16*). The collapse creates a catchment area with Pozzuoli acting as the ultimate drainage point. Celico *et al.* (*40*) identified an elevated hydraulic head gradient north of Pozzuoli (Fig. 4B, contour lines closer together showing a steep hydraulic gradient), which provides robust evidence for fluid circulation toward the lowest hydraulic point in Pozzuoli. Our analysis indicates a steady increase in the rate of change in rainfall over the past 24 years at both local rain gauges near Pozzuoli and regional gauges in the peri-Apennine mountains (Fig. 4A). We postulate that fluid accumulates over time, initially slowly, as suggested by the term bradyseism coined by Lyell (*5*), gradually filling the pore volume of the reservoir rock, controlling the magnitude of inflation. This process then accelerates, controlled by the rate of reservoir recharge, aligning with the observed rate of ground deformation (Fig. 2). We also show that the properties of hydrothermal water are crucial for the caprock’s self-healing through cementation, forming mineral fibers like those found in the natural CF caprock (*25*), thereby favoring ductile behavior and creeping processes.

Building on these observations, we propose a comprehensive mechanism in which fluid accumulation under hydrothermal conditions leads to pore pressure buildup, reducing effective stress. Upon fracturing, the rapid (isenthalpic) upflow of fluid, driven by liquid flashing to steam, can dynamically alter effective stress in the surrounding rocks, both vertically and laterally, ultimately facilitating the downward migration of seismicity.

Given the conditions for initiating rock failure (1) two primary stress-modification mechanisms have been identified as dominant triggers of earthquakes associated with reservoirs undergoing fluid filling (*53*): (i) increased pore pressure, which reduces effective normal stress; and (ii) direct changes in fault loading, where water-level fluctuations (e.g., reservoir depletion) decrease either normal stress or shear stress. In some cases, seismicity has been reported to occur only after multiple seasonal filling cycles have passed (*53*), suggesting a gradual diffusion of water from the reservoir, leading to accumulation at hypocentral depths. While these mechanisms were initially proposed for anthropogenic reservoir impoundment, the same fundamental processes are applicable to natural groundwater accumulation in a sealed reservoir, regardless of the water’s origin.

**Fig. 9.**
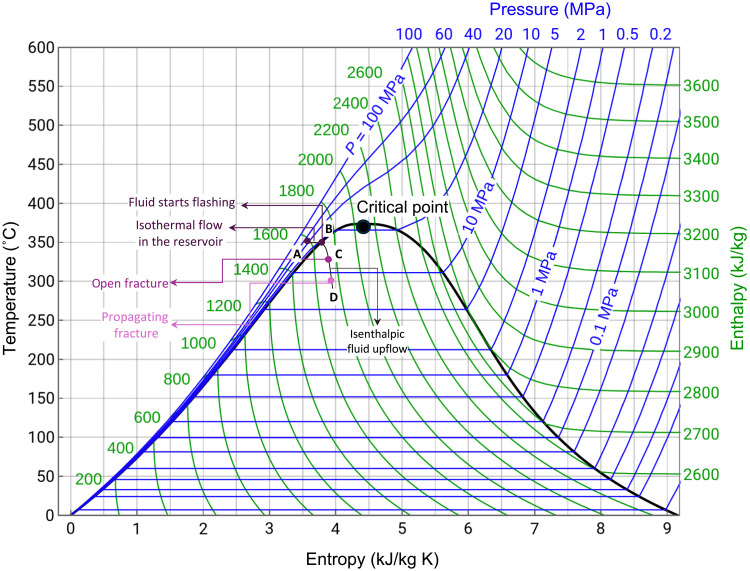
Temperature-entropy (*T*-*S*) diagram for water, showing constant enthalpy (green) and pressure (blue) lines. The phase envelope (black line) shows the saturated liquid (left of the critical point) and saturated vapor (right of the critical point) curves, with the critical point identified. The dark purple circle (A) marks the initial conditions (*T* = 350°C, *P* = 40 MPa) of the gas-enriched reservoir permeated by superheated water under the caprock. Before fracturing, the system is stable. When fracturing begins (B), the fluid starts flashing. As the fracture opens (C), it creates a rapid, nearly isenthalpic upflow of fluid. The ascent is accompanied by a sharp pressure drop, leading to evaporation (outgassing). As the fracture propagates from C to D, high-pressure fluid from the deep reservoir rushes toward shallower, lower-pressure regions. This drop in pore pressure increases effective stress, stabilizing the shallow fractures. Deeper parts remain under higher pore pressure if it cannot re-equilibrate throughout the different horizons. The pressure gradient between depressurized shallow regions and pressurized deeper zones drives a downward propagation of low effective stress, making deeper regions more prone to fracturing. As effective stress migrates downward, seismic activity follows suit.

We postulate that these stress-modification mechanisms progress at CFc through the following stages and processes:

1) Initial conditions of the system: The initial conditions of the proposed mechanism correspond to the stage just before the onset of microseismicity within the caprock seal, consistent with observations that early-stage unrest is marked by shallow seismic activity at depths of 1 to 2 km (Fig. 2). During this pre-failure stage (Fig. 9, point A), the gas/vapor-enriched reservoir is in equilibrium, with temperatures ranging from 300° to 350°C (fig. S1) (*20*, *21*) and pore fluid pressure nearing the minimum fracture strength of the caprock [45 MPa; (*25*)]. At these conditions, the water in the reservoir exists in a superheated liquid state (due to the PT conditions and maintains isothermal flow). The content of CO_2_ in the system (*20*, *21*) leads to a two-phase, two-component (gas-liquid) state (*20*, *21*), including vapor.

2) Fracture opening into the caprock and fluid upflow: As pore fluid pressure exceeds the minimum fracture strength of the caprock at the relevant depth (*25*), fracturing initiates, leading to the flashing of fluids (Fig. 9, point B). At this point, the two-phase fluid undergoes a slight increase in enthalpy (Fig. 9, points B and C) as heat from the surrounding hotter rocks is transferred to the cooler, boiling fluid. Rocks conduct heat to the adjacent fluid because of the temperature gradient between the rocks and the fluid. When the fluid boils (flashes to steam), it absorbs heat from its surroundings to support the phase change from liquid to vapor. This process requires an energy input called the latent heat of vaporization. While the increase in enthalpy is small, it indicates that heat from the rocks is transferred to the relatively cooler fluid, keeping the phase transition energetically supported. As fractures propagate (Fig. 9, point C), they (i) create pathways for rapid upward fluid escape, accelerating phase changes due to the pressure drop, and (ii) expose more surface area of the hot rocks to the fluid, enhancing heat transfer and maintaining the fluid’s enthalpy increase during the phase change.

3) Isenthalpic process and flashing to steam: The rapid upflow is nearly isenthalpic, with only minor decreases in enthalpy due to work against gravity and minimal heat loss. While the total heat content of the fluid remains nearly constant, its ascent is marked by a rapid pressure drop, leading to flash evaporation and outgassing. As the fracture continues to propagate (Fig. 9, points C and D), high-pressure fluids move swiftly toward shallower, lower-pressure regions. CO_2_ is released at pressures slightly higher than water vapor, given its higher vaporization threshold and greater solubility at depth. The rapid phase transition from liquid to vapor (flashing) leads to substantial fluid expansion, up to 1600 times, and a surge in kinetic energy, driving further fracture propagation. On one hand, the expanding steam exerts explosive pressure on the surrounding rock, intensifying fracturing and releasing stored stress. On the other, the abrupt volumetric increase due to sudden pressure release generates a shock wave, producing a loud, low-frequency sound that propagates through the ground and air. Earthquakes at CFs manifest themselves as deep rumbles akin to an underground explosion.

4) Pressure drop and effective stress redistribution: As fluid flashes and the system degasses (depletion of the geothermal reservoir), fluid pressure drops, triggering the observed migration of seismicity (*30*, *31*), both vertically and spatially. The rapid fluid upflow and subsequent phase change to steam relieve pressure in the shallower parts of the system, such as the caprock (Fig. 9, point D). This reduction in pore pressure increases the effective stress (σ_e_ = *P*_c_ − *P*_f_) in these shallow regions. As a result, the shallow rocks become less prone to further fracturing, stabilizing the upper portion of the system. In contrast, deeper regions may remain pressurized, forming a pore fluid pressure gradient that cannot readily equilibrate through fluid diffusion across the reservoir due to the low permeability of volcanoclastic siltite, where volcanic material (tuffites) clogs pore spaces, and the adjacent volcanic rocks (*24*, *25*, *30*). Previous authors (*54*) have reported a vapor-dominated plume characterized by relatively higher resistivity upwelling from a depth of 3 km through the caprock (*25*), before condensing beneath the Solfatara crater (lower resistivity zone).

5) Seismicity migration downward and laterally along caldera’s bordering faults: The pressure imbalance caused by the pore fluid pressure gradient between depressurized shallow zones and pressurized deeper regions, exacerbated by the low permeability of volcanic rocks, leads to reduced effective stress in deeper regions. Additionally, as upwelling fluid discharge and degassing occur through the caprock, the resulting unloading further reduces normal stress (σ_v_) on the deeper zones. Together, these processes induce a dynamic redistribution of effective stress, progressively driving the migration of seismic activity to greater depths over time. Recent studies (*30*, *31*) indicate that the deepest seismicity first begins in the western part of the caldera, offshore toward the Baia-Bacoli coastline. This area may also serve as the deepest point where water drains and collects as limestone dip toward the center of the bay of Pozzuoli (*14*, *16*, *30*). As degassing progresses and the reservoir continues to deplete, the load on the surrounding (lateral) rocks decreases. This reduction in pressure lowers the horizontal component of the normal stress along the bordering faults, which, in turn, decreases critical shear stress. Over time, this leads to fault slip, triggering seismic activity in the abovementioned western part of the caldera before it later shifts to the eastern part, offshore toward the Bagnoli coastline (*30*, *31*).

The results from this study, the geophysical observations during the unrest, and the proposed mechanism suggest that, hydraulically, the cemented caprock acts as a barrier (see movies S1 and S2) (*25*), preventing the upward migration of fluids such as water, steam, or CO_2_ (*12*, *32*). Mechanically, it functions as a throttle valve (*55*), closing through cementation and high effective stress while opening through fracturing as pore fluid pressure increases lowering effective stress. Ultimately, that provides a pressure release mechanism (*56*) that let fluid escape, eventually leading to subsidence. In this context, hydrothermal fluids play a dual role: They serve as chemical agents during cementation and pressure depletion, as well as physical agents, reducing effective normal stress through rapid isenthalpic upflow, which leads to fracturing.

The steady temperature decrease of the main fumarole (Pisciarelli) since 2018 [figure 5.1 of (*57*)] suggests a continuous influx of colder water into the subsurface. Additionally, there is no recorded deep seismicity between the melt region at 7- to 8-km depth and 5 km. If melt were rising, then it would influence fumarole temperatures and trigger seismic activity, suggesting that it is either not ascending or not directly involved in the ongoing unrest. Under this scenario, the deep heat source (*17*) seems to primarily act as the burner of the geothermal system, supplying energy to the fuel, the fluids, that sustain hydrothermal processes and thermometamorphic reactions. As this fuel depletes through continuous fracturing and outgassing, pore fluid pressure decreases slowly leading to deflation (Fig. 7, open red circles) (subsidence). If magma had ascended or hot bodies had intruded to explain the uplift, then it is unclear where these masses would go to allow a subsidence of 90 cm since 1985. Once emplaced, dykes and sills occupy solid mass and volume. For subsidence to occur, mass must leave the porous medium, which can only be explained by mass exiting the system as fluids, whether vapor or gases, or liquid water.

### Implications for risk management and engineering solutions

The proposed mechanism aligns with the geophysical observations from the two most recent unrest episodes and historical accounts of the last eruption in 1538, which formed the cinder cone of Mt. Nuovo (*13*, *58*). Chronicles from the time describe how, in the hours preceding the eruption, inhabitants witnessed the formation of fractures across the bulging ground, followed by an abundant outpouring of cold, and then hot, fresh water. The eruption itself was characterized by intense groundwater involvement, producing vapor-charged white clouds and the downwind fallout of muddy ash over Pozzuoli. This description highlights the fluid-dynamic instability underlying phreatic explosions (*59*), such as the recent eruption of the San Jose Mulatos mud volcano in Colombia. This instability propagates through sudden fracturing, rapid fluid upflow, and the violent expulsion of boiling mud and ash, during which a catalytic hydrogenation reaction occurs. Here, carbon dioxide (CO_2_) reacts with hydrogen (H_2_), the latter formed at high temperatures through thermochemical water splitting or steam reforming, to produce methane (CH_4_) and water (H_2_O). The released methane can ignite and flare, further intensifying the explosivity of these events and adding to their abrupt and hazardous nature.

Unlike medical imaging, where the recurrence of anomalies is closely linked to well-defined anatomy and established physiological functions, subsurface environments are far more variable and dynamic, making them inherently less predictable. Recent activity at Campi Flegrei has prompted the scientific community to better understand the ongoing unrest in comparison to the 1982–1984 crisis, an event that led to the establishment of a continuous monitoring system, which has become more efficient and advanced over time (*60*). Detecting the recurrence of anomalies within the structure of the CFc and proposing a mechanism that reconciles cross-disciplinary observations marks a breakthrough in understanding the unrest of this complex volcanic-geothermal system, one that functions both as a volcanic field and an active hydrodynamic system. This has profound implications for hazard assessment and risk management.

Phreatic eruptions, while often short-lived, are highly unpredictable and can be extremely dangerous for nearby populations. These eruptions can occur with little warning, as they are driven by the instantaneous flashing of water to steam when heated by underlying magma. The explosive nature of these eruptions, resulting from steam expansion, presents highly localized but high-impact hazards, from shock waves from rapid steam expansion to ballistic projectiles and ashfall, to landslides and sinkholes, particularly in unstable hydrothermal zones.

Unlike large-scale magmatic eruptions, phreatic events may not require mass evacuations of entire regions but rather may necessitate localized evacuations near active vents, crater lakes, or hydrothermal fields. In addition to monitoring fumarole temperatures (including cooling that can indicate instability) and gas composition, tracking the widespread formation of sinkholes along with groundwater levels provides key indicators of changing conditions in the system. Given that phreatic eruptions are primarily driven by hydrothermal dynamics, authorities must place particular emphasis on monitoring groundwater levels and pressure in wells. Monitoring groundwater levels is critical. It offers the most direct insight into subsurface fluid dynamics and allows hazard management to shift from reactive crisis monitoring to proactive long-term forecasting, enabling authorities to identify impending risks well before they fully manifest through seismic activity.

The mechanism proposed in this study provides actionable insights not only for hazard assessment but also for engineering solutions that support risk reduction strategies. It emphasizes the need for proactive monitoring of key parameters and response teams, not just evacuation plans. As part of this, we propose an engineering solution consisting in intercepting water upstream before it drains toward Pozzuoli, such as managing anthropic channels regulating water flow and/or withdrawing water from existing wells. This solution aims to reduce the hazards in an area that has been affected by such phenomena for millennia, ultimately mitigating the cyclical financial and social burden on the affected community of Pozzuoli.

## MATERIALS AND METHODS

### Seismic catalog

The catalog contains ~12,681 seismic events (*30*) recorded between August 2014 and November 2024 by the 21 stations of the National Institute of Geophysics and Volcanology (INGV) network. Using P and S arrival times and magnitude estimates from the INGV earthquake catalog (https://terremoti.ov.ingv.it/gossip/flegrei), we found that event magnitudes (*M*_d_) ranged from −1.1 to 4.4, with most between 0 and 1. On average, each event had seven to eight P and two to three S arrival times, with the largest earthquake (*M*_d_ 4.4, 20 May 2024, at 18:10 UTC) having up to 15 P and 6 S arrival times.

The events are located using a nonlinear probabilistic location method [NLLoc-SSST; (*61*, *62*)] within the one-dimensional velocity model of Vanorio *et al.* (*15*). This method offers two main advantages: (i) It reconstructs the posterior probability density function, providing a comprehensive assessment of location uncertainties; and (ii) it integrates SSST corrections, greatly improving relative location accuracy by reducing errors due to velocity model uncertainties.

The final catalog from (*30*) consists of ~10,500 high-quality event locations, with root mean square errors below 0.1 s, a maximum azimuthal gap within 200°, and horizontal and vertical location errors within 500 m for 98% of events and within 800 m for the remaining 2%. The final catalog events used for the tomographic inversion with 75,056 P-picks and 29,267 S-picks. Notably, 82% of events in the final catalog have more than eight total phases, 91% have at least two S-picks, and 60% have more than three S-picks, reducing cross-talk between velocity and location perturbations.

### Ground deformation data

We extracted the vertical displacement from global navigation satellite system (GNSS) measurement from January 2000 to May 2024 taken at RITE (Rione Terra-Pozzuoli), station of the permanent INGV RING (Rete Integrata Nazionale GNSS; https://webring.gm.ingv.it/) network. The data processing, provided by INGV Osservatorio Vesuviano surveillance team (*63*), included the removal of an annual and a semiannual trend, and the removal of the background regional tectonic pattern, extracted by six stations of the INGV RING network located outside the Neapolitan volcanic area (*63*). The data from 25 INGV RING stations in Campi Flegrei area (including four stations off-shore the Pozzuoli Bay), from 2011 to 2019, was interpolated to construct the vertical deformation pattern that we represented in Fig. 3 (right) (*63*).

### Pluviometry and piezometric data

We accessed the precipitation data catalog for the rain gauges shown in fig. S2 through the Civil Protection of the Campania Region (*39*) (https://centrofunzionale.regione.campania.it/-/pages/sensori/archivio-pluviometrici).

We extracted daily estimates from 2000 to 2023 and calculated the total annual precipitation. Then, we determined the year-to-year percentage change using the following formula

$$\begin{array}{c} \text{Percentage change}\;\left(\text{Year}\;X\right)\\ =\frac{\text{Annual estimate}\;\left(\text{Year}\;X\right)-\text{Annual estimate}\;\left(\text{Year}\;X-1\right)}{\text{Annual estimate}\;\left(\text{Year}\;X-1\right)} \end{array}$$

The piezometric head data were obtained from Agenzia Regionale Protezione Ambientale della Campania (ARPAC)’s Monitoring System of Subsurface Water and supplemented by data from (*41*). This combined dataset included measurements from 530 wells, with a grid of 61 wells selected based on homogeneity criteria.

### Cementation experiment

The cementation experiment simulates the subsurface conditions of the CFc, focusing on temperature, fluid type, and composition. The process is detailed as follows (fig. S4): A funnel-shaped filter (fig. S4A) is sealed on top of a hollow Teflon cylinder (fig. S4, B and C). Unconsolidated material simulating caprock composition is poured into the funnel (fig. S4E), with filter paper used to prevent material loss through the holes (fig. S4D). A fine mesh steel filter is placed above the material to avoid spillage (fig. S4F), and the entire assembly is sealed with Teflon tape (fig. S4G). The setup is then placed in a Parr reactor (450-ml capacity; Fig. 5C) and maintained at 200°C for 24 hours. After natural cooling, the cemented material (fig. S4H) is removed from the funnel and dried in an oven.

To mimic caprock formation, a trachyte-phonolite volcanic rock was mixed with a small amount of lime (CaO), calcined at 1100°C for 1.5 hours in a Thermo Scientific Lindberg Blue M furnace, and finely ground using a SPEX Shatterbox. During the experiment, the hollow Teflon cylinder was immersed in a saturated solution of CaO (limewater). After drying, scanning electron microscopy analysis was conducted on the cemented material. The samples were mounted on metal stubs, coated with carbon using a Leica EM ACE 600 high vacuum sputter coater, and analyzed with a JEOL JSM-IT500HT scanning electron microscope in high vacuum mode, with an accelerating voltage between 10 and 15 kV and a probe current of 50 to 56 nA. Images are saved at a resolution of 1016 dots per inch (dpi).

### Rock physics experiment

Porosity of the sample was measured using a helium porosimeter based on Boyle’s law to determine grain and solid volume. Bulk density was calculated by measuring the sample’s mass and volume. These properties served as baseline reference points before subjecting the sample to stress.

P-wave and S-wave velocities, as well as bulk volume changes in response to pore fluid pressure and flow rate, were measured using a custom-built acoustic pressure vessel. This system includes a core holder, three linear potentiometers, a pulse generator (Avtech AVR-7B-B), a switch (Hewlett Packard 4388A), and a digital oscilloscope (Tektronix TDS 1012B). For further details on the acoustic vessel system, see Stanford Acoustic System (https://srpvl.stanford.edu/Acoustic_System/).

Although change in velocities is not the focus of the paper, real-time videos showing changes in recorded waveforms are provided as part of the Supplementary Materials to show how velocity decreases (i.e., travel time increases) and waveform amplitude decreases as pore pressure increases in the porous rock.

Samples were initially dry and enclosed in rubber tubing to protect them from the confining pressure medium (oil). Confining pressure (*P*_c_) was set at 25 MPa and maintained 0.5 MPa higher than the pore fluid pressure (*P*_f_) to prevent jacket leakage. The sample jacket was equipped with radial clamps to prevent fluid leakage. Each end plate of the core holder featured a pore fluid inlet for fluid passage through the sample. The pore pressure lines were connected to a gas-driven liquid pump (2.2 HP Haskel) for fluid injection. A flow meter was installed upstream to measure the injection rate, while pressure gauges were positioned upstream and downstream along the pore fluid line. Three linear potentiometers measured strain due to stress, which was used to estimate bulk volume and porosity changes relative to the baseline, assuming pore closure as the primary strain mechanism.

## References

[1] D. P. Hill, Long Valley Caldera–Mammoth Mountain unrest: The knowns and the unknowns. Elements 13, 8–9 (2017).

[2] F. Barberi, G. Corrado, F. Innocenti, G. Luongo, Phlegraean Fields 1982–1984: brief chronicle of a volcano emergency in a densely populated area. Bull. Volcanol. 47, 175–185 (1984).

[3] D. P. Hill, Unrest in Long Valley Caldera, California, 1978–2004. Geol. Soc. Lond. Spec. Publ. 269, 1–24 (2006).

[4] C. Del Gaudio, I. Aquino, G. P. Ricciardi, C. Ricco, R. Scandone, Unrest episodes at Campi Flegrei: A reconstruction of vertical ground movements during 1905–2009. J. Volcanol. Geotherm. Res. 195, 48–56 (2010).

[5] C. Lyell, *Principles of Geology: An Attempt to Explain the Former Changes of the Earth’s Surface* (Cambridge Univ. Press, 1830), vol. 1.

[6] G. Luongo, E. Cubellis, F. Obrizzo, S. M. Petrazzuoli, The mechanics of the Campi Flegrei resurgent caldera —A model. J. Volcanol. Geotherm. Res. 45, 161–172 (1991).

[7] L. De Siena, A. Amoruso, E. Del Pezzo, Z. Wakeford, M. Castellano, L. Crescentini, Space-weighted seismic attenuation mapping of the aseismic source of Campi Flegrei 1983–1984 unrest. Geophys. Res. Lett. 44, 1740–1748 (2017).

[8] L. De Siena, E. Del Pezzo, F. Bianco, Seismic attenuation imaging of Campi Flegrei: Evidence of gas reservoirs, hydrothermal basins, and feeding systems. J. Geophys. Res. 115, 10.1029/2009JB006938 (2010).

[9] M. Calò, A. Tramelli, Anatomy of the Campi Flegrei caldera using enhanced seismic tomography models. Sci. Rep. 8, 16254 (2018). 30389977 10.1038/s41598-018-34456-x PMC6214947

[10] G. Giacomuzzi, C. Chiarabba, F. Bianco, P. De Gori, N. P. Agostinetti, Tracking transient changes in the plumbing system at Campi Flegrei Caldera. Earth Planet. Sci. Lett. 637, 118744 (2024).

[11] L. Casertano, A. O. del Castillo, M. T. Quagliariello, Hydrodynamics and geodynamics in the Phlegraean Fields area of Italy. Nature 264, 161–164 (1976).

[12] G. Chiodini, S. Caliro, C. Cardellini, D. Granieri, R. Avino, A. Baldini, M. Donnini, C. Minopoli, Long-term variations of the Campi Flegrei, Italy, volcanic system as revealed by the monitoring of hydrothermal activity. J. Geophys. Res. Solid Earth 115, 10.1029/2008JB006258 (2010).

[13] J. J. Dvorak, P. Gasparini, History of earthquakes and vertical ground movement in Campi Flegrei caldera, Southern Italy: comparison of precursory events to the AD 1538 eruption of Monte Nuovo and of activity since 1968. J. Volcanol. Geotherm. Res. 48, 77–92 (1991).

[14] S. Judenherc, A. Zollo, The Bay of Naples (southern Italy): Constraints on the volcanic structures inferred from a dense seismic survey. J. Geophys. Res. 109, 10.1029/2003JB002876 (2004).

[15] T. Vanorio, J. Virieux, P. Capuano, G. Russo, Three-dimensional seismic tomography from P wave and S wave microearthquake travel times and rock physics characterization of the Campi Flegrei Caldera. J. Geophys. Res. 110, 10.1029/2004JB003102 (2005).

[16] A. Milia, M. M. Torrente, “Space-time evolution of an active volcanic field in an extensional region: The example of the Campania margin (eastern Tyrrhenian Sea)” in *Vesuvius, Campi Flegrei, and Campanian Volcanism*, B. De Vivo, H. Belkin, G. Rolandi, Eds. (Elsevier, ed. 1, 2020), pp. 297–321.

[17] A. Zollo, N. Maercklin, M. Vassallo, D. D. Iacono, J. Virieux, P. Gasparini, Seismic reflections reveal a massive melt layer feeding Campi Flegrei caldera. Geophys. Res. Lett. 35, 10.1029/2008GL034242 (2008).

[18] R. C. Aster, R. P. Meyer, Three-dimensional velocity structure and hypocenter distribution in the Campi Flegrei caldera, Italy. Tectonophysics 149, 195–218 (1988).

[19] C. Chiarabba, M. Moretti, An insight into the unrest phenomena at the Campi Flegrei caldera from Vp and Vp/Vs tomography. Terra Nova 18, 373–379 (2006).

[20] AGIP, “Modello Geotermico del Sistema Flegreo” (Report, AGIP SERG-MESG, 1987).

[21] R. Carella, M. Guglielminetti, “Multiple reservoirs in the Mofete field, Naples, Italy” (SGP-TR-74, Stanford University, 1983); https://pangea.stanford.edu/ERE/pdf/IGAstandard/SGW/1983/Carella.pdf.

[22] B. De Vivo, H. E. Belkin, M. Barbieri, W. Chelini, P. Lattanzi, A. Lima, L. Tolomeo, The Campi Flegrei (Italy) geothermal system: A fluid inclusion study of the Mofete and San Vito fields. J. Volcanol. Geotherm. Res. 36, 303–326 (1989).

[23] M. Zamora, G. Sartoris, W. Chelini, Laboratory measurements of ultrasonic wave velocities in rocks from the Campi Flegrei volcanic system and their relation to other field data. J. Geophys. Res. 99, 13553–13561 (1994).

[24] T. Vanorio, M. Prasad, D. Patella, A. Nur, Ultrasonic velocity measurements in volcanic rocks: Correlation with microtexture. Geophys. J. Int. 149, 22–36 (2002).

[25] T. Vanorio, W. Kanitpanyacharoen, Rock physics of fibrous rocks akin to Roman concrete explains uplifts at Campi Flegrei Caldera. Science 349, 617–621 (2015). 26160377 10.1126/science.aab1292

[26] V. C. Li, H. Stang, Interface property characterization and strengthening mechanisms in fiber reinforced cement-based composites. Adv. Cem. Based Mater. 6, 1–20 (1997).

[27] A. Valadez-Gonzalez, J. M. Cervantes-Uc, R. Olayo, P. J. Herrera-Franco, Effect of fiber surface treatment on the fiber–matrix bond strength of natural fiber reinforced composites. Compos. Part B Eng. 30, 309–320 (1999).

[28] L. Mezeix, C. Bouvet, J. Huez, D. Poquillon, Mechanical behavior of entangled fibers and entangled cross-linked fibers during compression. J. Mater. Sci. 44, 3652–3661 (2009).

[29] T. Vanorio, J. Chung, S. Siman-Tov, A. Nur, Hydrothermal formation of fibrous mineral structures: The role in strength and mode of failure. Front. Earth Sci. 10, 1052447 (2023).

[30] G. De Landro, T. Vanorio, T. Muzellec, G. Russo, J. Virieux, A. Lomax, F. Scotto di Uccio, G. Festa, A. Zollo, “Unveiling the Campi Flegrei inner caldera structure from 3D high-resolution local earthquake tomography imaging” in *39th General Assembly of the European Seismological Commission* (European Seismological Commission, 2024).

[31] F. Scotto di Uccio, A. Lomax, J. Natale, T. Muzellec, G. Festa, S. Nazeri, V. Convertito, A. Bobbio, C. Strumia, A. Zollo, Delineation and fine-scale structure of fault zones activated during the 2014–2024 unrest at the Campi Flegrei caldera (Southern Italy) from high-precision earthquake locations. Geophys. Res. Lett. 51, e2023GL107680 (2024).

[32] M. Todesco, Caldera’s breathing: Poroelastic ground deformation at Campi Flegrei (Italy). Front. Earth Sci. 9, 702665 (2021).

[33] I. Iervolino, P. Cito, M. de Falco, G. Festa, M. Herrmann, A. Lomax, W. Marzocchi, A. Santo, C. Strumia, L. Massaro, A. Scala, F. Scotto di Uccio, A. Zollo, Seismic risk mitigation at Campi Flegrei in volcanic unrest. Nat. Commun. 15, 10474 (2024). 39622825 10.1038/s41467-024-55023-1 PMC11612255

[34] H. Ito, J. DeVilbiss, A. Nur, Compressional and shear waves in saturated rock during water-steam transition. J. Geophys. Res. 84, 4731–4735 (1979).

[35] G. M. Mavko, Velocity and attenuation in partially molten rocks. J. Geophys. Res. Solid Earth 85, 5173–5189 (1980).

[36] C. B. Raleigh, J. H. Healy, J. D. Bredehoeft, An experiment in earthquake control at Rangely, Colorado. Science 191, 1230–1237 (1976). 17737698 10.1126/science.191.4233.1230

[37] M. K. Hubbert, W. W. Rubey, Role of fluid pressure in mechanics of overthrust faulting. Geol. Soc. Am. Bull. 70, 115–206 (1959).

[38] G. Chiodini, M. Todesco, S. Caliro, C. D. Gaudio, G. Macedonio, M. Russo, Magma degassing as a trigger of bradyseismic events: The case of Phlegraean Fields (Italy). Geophys. Res. Lett. 30, 1434 (2003).

[39] Ance Campania, Pluviometric data. www.ancecampania.it/category/dati-pluviometrici/.

[40] P. Celico, P. De Vita, F. Nikzad, D. Stanzione, A. Vallario, “Schema idrogeologico e idrogeochimico dei Campi Flegrei (NA)” in *Atti I Convegno Nazionale dei Giovani Ricercatori in Geologia Applicata* (Università degli studi di Milano Supplemento, 1991), pp. 287–296.

[41] ARPAC, Monitoring System of Subsurface Water. www.webcampania.com/.

[42] V. Allocca, L. Castellucci, S. Coda, M. Coromaldi, P. De Vita, E. Marzano, Integrating hydrogeological and economic analyses of groundwater flooding in an urban aquifer: the plain of Naples (Italy) as a case study. Int. J. Environ. Stud. 80, 1400–1416 (2023).

[43] G. Chiodini, L. Pappalardo, A. Aiuppa, S. Caliro, The geological CO_2_ degassing history of a long-lived caldera. Geology 43, 767–770 (2015).

[44] D. Head, T. Vanorio, A. C. Clark, Elastic softening of limestone upon decarbonation with episodic CO_2_ release. J. Geophys. Res. Solid Earth 123, 7404–7420 (2018).

[45] C. Panichi, E. Tongiorgi, “Carbon isotopic composition of CO2 from springs, fumaroles, mofettes, and travertines of central and southern Italy. Preliminary prospection method of a geothermal area” in *Proceedings of the Second United Nations Symposium on the Development and Use of Geothermal Resources* (US Government Printing Office, 1976), pp. 815–825.

[46] G. Buono, S. Caliro, A. Paonita, L. Pappalardo, G. Chiodini, Discriminating carbon dioxide sources during volcanic unrest: The case of Campi Flegrei caldera. Geology 51, 397–401 (2023).

[47] J. P. Gratier, P. Favreau, F. Renard, E. Pili, Fluid pressure evolution during the earthquake cycle controlled by fluid flow and pressure solution crack sealing. Earth Planets Space 54, 1139–1146 (2002).

[48] M. M. Unterlass, Geomimetics and extreme biomimetics inspired by hydrothermal systems—What can we learn from nature for materials synthesis? Biomimetics 2, 8 (2017). 31105171 10.3390/biomimetics2020008 PMC6477620

[49] Q. Y. Wang, X. Cui, W. B. Frank, Y. Lu, T. Hirose, K. Obara, Untangling the environmental and tectonic drivers of the Noto earthquake swarm in Japan. Sci. Adv. 10, eado1469 (2024). 38718113 10.1126/sciadv.ado1469 PMC11078177

[50] N. Scafetta, A. Mazzarella, On the rainfall triggering of Phlegraean Fields volcanic tremors. Water 13, 154 (2021).

[51] L. De Siena, A. Amoruso, S. Petrosino, L. Crescentini, Geophysical responses to an environmentally-boosted volcanic unrest. Geophys. Res. Lett. 51, e2023GL10489 (2024).

[52] F. Giudicepietro, G. Chiodini, R. Avino, G. Brandi, S. Caliro, W. de Cesare, D. Galluzzo, A. Esposito, A. la Rocca, D. Lo Bascio, F. Obrizzo, S. Pinto, T. Ricci, P. Ricciolino, A. Siniscalchi, A. Tramelli, J. Vandemeulebrouck, G. Macedonio, Tracking episodes of seismicity and gas transport in Campi Flegrei Caldera through seismic, geophysical, and geochemical measurements. Seismol. Res. Lett. 92, 965–975 (2021).

[53] D. W. Simpson, W. S. Leith, C. H. Scholz, Two types of reservoir-induced seismicity. Bull. Seismol. Soc. Am. 78, 2025–2040 (1988).

[54] A. Siniscalchi, S. Tripaldi, G. Romano, G. Chiodini, L. Improta, Z. Petrillo, L. D'Auria, S. Caliro, R. Avino, Reservoir structure and hydraulic properties of the Campi Flegrei geothermal system inferred by audiomagnetotelluric, geochemical, and seismicity study. J. Geophys. Res. Solid Earth 124, 5336–5356 (2019).

[55] S. A. Wood, F. J. Spera, Adiabatic decompression of aqueous solutions: Applications to hydrothermal fluid migration in the crust. Geology 12, 707–710 (1984).

[56] R. H. Sibson, Conditions for fault-valve behavior. Geol. Soc. Lond. Spec. Publ. 54, 15–28 (1990).

[57] Vesuvian Observatory, Campi Flegrei Weekly Bulletin, 11 June 2024. www.ov.ingv.it/index.php/monitoraggio-e-infrastrutture/bollettini-tutti/boll-sett-flegrei/anno-2024/1647-bollettino-flegrei-2024-06-11/file.

[58] C. D’Oriano, E. Poggianti, A. Bertagnini, R. Cioni, P. Landi, M. Polacci, M. Rosi, Changes in eruptive style during the A.D. 1538 Monte Nuovo eruption (Phlegraean Fields, Italy): The role of syn-eruptive crystallization. Bull. Volcanol. 67, 601–621 (2005).

[59] C. Montanaro, E. Mick, J. Salas-Navarro, C. Caudron, S. J. Cronin, J. M. de Moor, B. Scheu, J. Stix, K. Strehlow, Phreatic and hydrothermal eruptions: From overlooked to looking over. Bull. Volcanol. 84, 64 (2022).

[60] S. Danesi, N. A. Pino, S. Carlino, C. R. J. Kilburn, Evolution in unrest processes at Campi Flegrei caldera as inferred from local seismicity. Earth Planet. Sci. Lett. 626, 118530 (2024).

[61] A. Lomax, A. Michelini, A. Curtis, Earthquake location, direct global-search methods. Encyclop. Complex. Syst. Sci. 5, 2449–2473 (2009).

[62] A. Lomax, A. Savvaidis, High-precision earthquake location using source-specific station terms and inter-event waveform similarity. J. Geophys. Res. Solid Earth 127, e2021JB023190 (2022).

[63] P. De Martino, M. Dolce, G. Brandi, G. Scarpato, U. Tammaro, The ground deformation history of the Neapolitan volcanic area (Campi Flegrei caldera, Somma–Vesuvius volcano, and Ischia Island) from 20 years of continuous GPS observations (2000–2019). Remote Sens. 13, 2725 (2021).

[64] S. Vitale, S. Ciarcia, Tectono-stratigraphic setting of the Campania region (southern Italy). J. Maps 14, 9–21 (2018).

[65] J. Natale, G. Camanni, L. Ferranti, R. Isaia, M. Sacchi, V. Spiess, L. Steinmann, S. Vitale, Fault systems in the offshore sector of the Campi Flegrei caldera (southern Italy): Implications for nested caldera structure, resurgent dome, and volcano-tectonic evolution. J. Struct. Geol. 163, 104723 (2022).

