# Supplementary material for: The recurrence of geophysical manifestations at the Campi Flegrei caldera
Source: Sci Adv. 2025 May 2;11(18):eadt2067. doi: 10.1126/sciadv.adt2067 (PMC12047431; doi:10.1126/sciadv.adt2067)
Supplement: Supplementary file 1 — Supplementary Text S1 Figs. S1 to S4 Legends for movies S1 and S2 [file sciadv.adt2067_sm.pdf]

Science Advances
AAAS

Supplementary Materials for

The recurrence of geophysical manifestations at the Campi Flegrei caldera

Tiziana Vanorio *et al.*

Corresponding author: Tiziana Vanorio, tvanorio@stanford.edu

Sci. Adv. **11**, eadt2067 (2025)
DOI: 10.1126/sciadv.adt2067

The PDF file includes:

Supplementary Text S1
Figs. S1 to S4
Legends for movies S1 and S2

Other Supplementary Material for this manuscript includes the following:

Movies S1 and S2

Text S1: Pore fluid pressure CO_2-generating decarbonation reactions

Reaction (1) indicates that for every mole of calcite in limestone reacting with a mole of water, one mole of CO_2 is produced along with half a mole of wollastonite, lime, and water. Considering molar masses, 180 kg of H_2O reacts with 1000 kg of calcite to produce 440 kg of CO_2.

Let us assume that 1 m^3 of water enters the caldera, thus producing 2444.44 kg of CO_2 due to decarbonation, we need to calculate the pressure exerted by this CO_2. At a depth of 3 km in the CF caldera, with a temperature more than 300°C, CO_2 is supercritical. The critical pressure of CO_2 is 7.38 MPa, and the critical temperature is 31°C. First, the molar mass of CO_2 is 44.01 g/mol. Converting 2444 kg of CO_2 to moles gives:

$$\text{moles of } CO_2 = \frac{2444\ kg}{44.01\ g/mol} = 55539.64\text{mol}$$

Using the ideal gas law for an approximation, the molar volume of supercritical CO_2 at 300°C (573.15 K) is calculated as:

$$V_m = \frac{8.314\frac{J}{mol}\cdot K\ x(300+273.15)K}{P} \approx \frac{8314\ x\ 573.15}{P} \approx \frac{4761.24}{P}\ m^3/mol$$

The total volume occupied by 2444 kg of CO_2 is:

$$V_{total} = \text{moles of } CO_2 \times V_m$$

$$\text{Moles of } CO_2 = \frac{2444\ kg}{44.01\ g/mol} = 55539.64\ \text{mol}$$

$$V_{total} = 55539.64\ \text{mol x } \frac{4761.24}{P}\ m^3/mol \approx \frac{264634673.28}{P}\ m^3$$

Finally, using the ideal gas law to find the pressure (P):

$$P = \frac{nRT}{V_{total}} \approx \frac{55539.64\ x\ 8.314\ x\ 573.15}{\frac{264634673.28}{P}} \approx \frac{2.30948\ x\ 10^8}{\frac{264634673.28}{P}} \approx 57.43\ MPa$$

Therefore, the pressure exerted by 2444 kg of supercritical CO_2 at 300°C is approximately 57.43 MPa.

At 300°C, the saturation pressure of water is approximately 8.5 MPa. This high pressure reflects the state of water as a supercritical fluid at this temperature, where the properties of water and steam merge. This value is obtained from standard steam tables and aligns with calculations based on the Clausius-Clapeyron equation for water's vapor pressure.

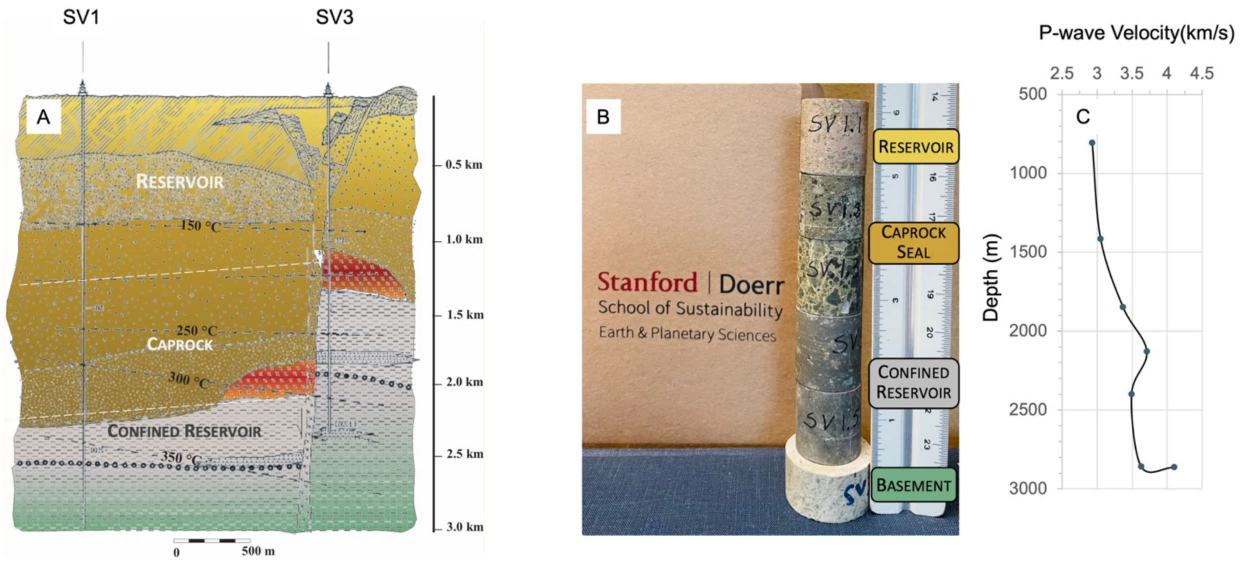

Fig. S1. Geological section and stratigraphy of the CFc. (A) Lithostratigraphic units are reconstructed from wells SV3 and SV1, modified after (20). (B) Core samples from the SV1 well, stacked one on top of the other, to reconstruct the stratigraphy in (A) and provide a clear visual of the geological layers along their P-wave velocity (C). Drilling has revealed multiple reservoirs: a shallow, water-dominated reservoir up to 1 km depth within a tuff formation, and a deeper, gas-enriched reservoir located below approximately 2 km within a volcaniclastic sandstone—a consolidated marine siltite-sand mixed with volcanic material (tuffites). These reservoirs are separated by an impermeable layer, a fiber-rich caprock seal from the pozzolanic cementation of pyroclastic material (ignimbrite) whose microstructure is characterized by (25). Studies using seismic tomography have seismically characterized the shallow reservoir by high Vp/Vs ratios (8, 15, 18-19), while the reservoir at depths below 2 km by low Vp/Vs ratios (8, 15, 19), indicative of gas enrichment and overpressure. This confined reservoir transitions into the basement, a metamorphosed siliciclastic limestone containing a mineral paragenesis indicative of decarbonation reactions (25).

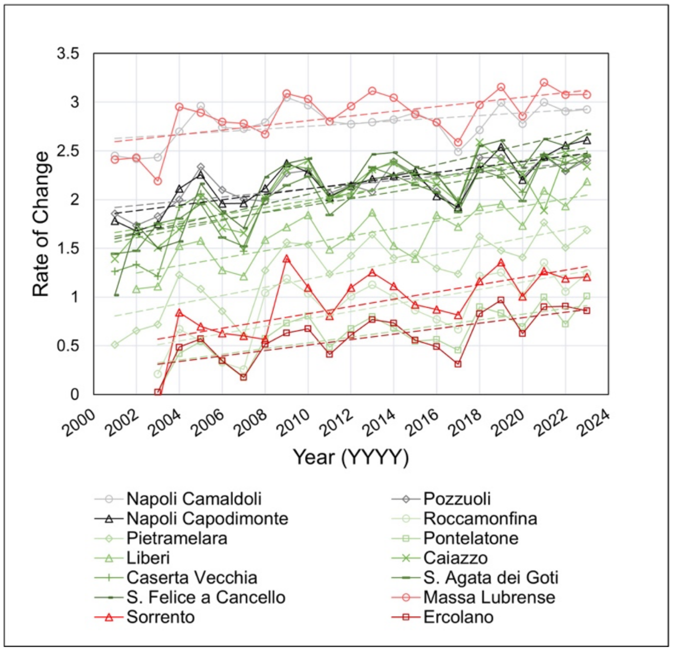

Fig. S2. Change of rainfall as a function of time for all rain gauge stations analyzed and shown in fig. S1. Different colors represent different geographical areas: black-grey shades for Pozzuoli, green shades for the Peri- and Apennine areas, and red shades for the Sorrento Peninsula and Vesuvian Area.

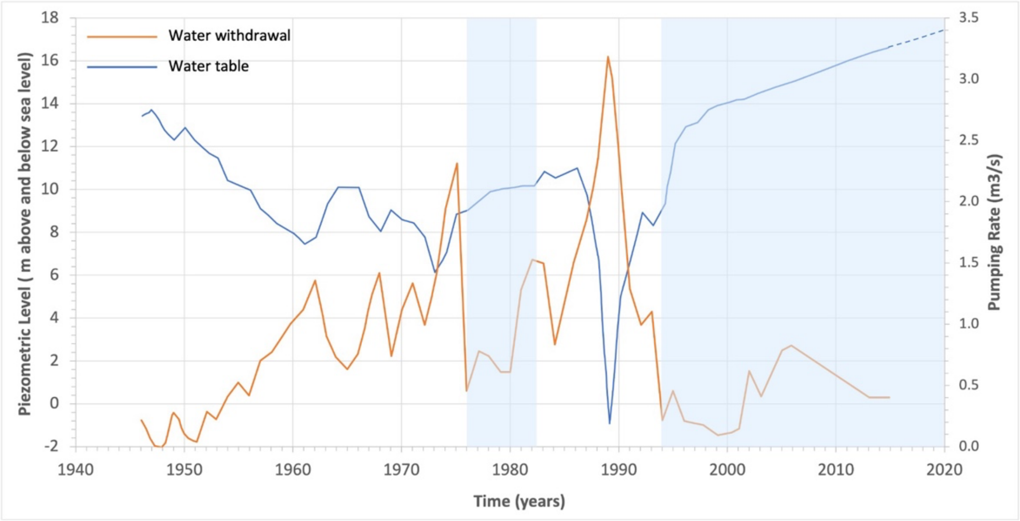

Fig. S3 Time series of groundwater levels (blue) and withdrawal pumping rates (orange) for the Lufrano well-field (blue mark in Fig. 1) in the Neapolitan area. Light blue shading highlights water head fluctuations and withdrawal pumping rates over the past 24 years, including the 1982-1984 period.

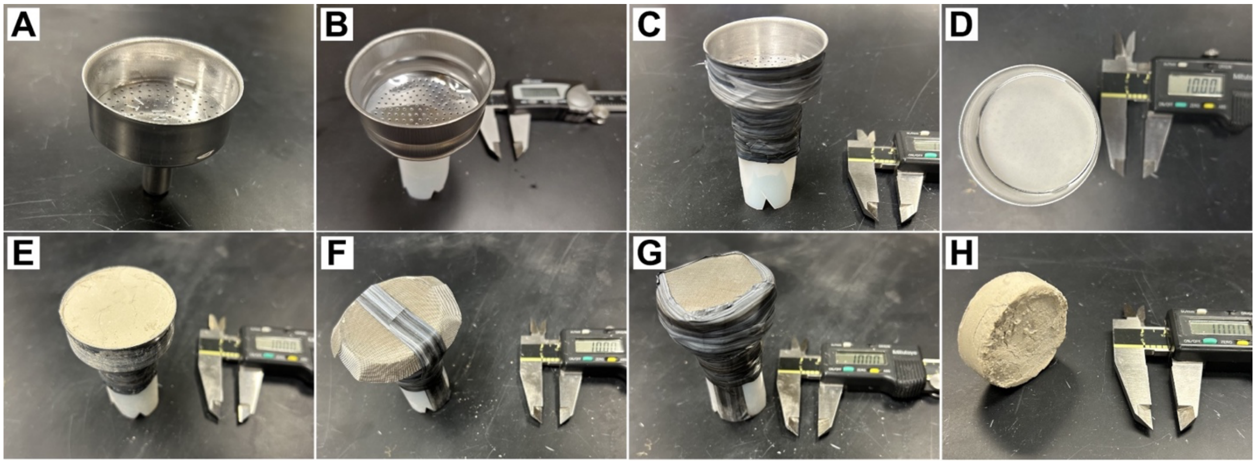

Fig. S4. Step sequence of the cementation experiment to simulate the subsurface conditions of caprock formation. From A to G, it shows the creation of the custom-made assembly, which is placed inside the Parr reactor (Fig.5, C) at 200˚C and autogenous pressure, leading to the formation of a cemented fibrous material (H) whose microstructure is shown in Fig.6.

M1: Full P-wave waveform displaying real-time variations in amplitude and arrival time as a function of pore fluid pressure. These dynamic changes result from pore fluid buildup, diffusion and the re-equilibration of pore pressure throughout the sample.

M2: Zoomed-in view of full P-wave waveform, displaying real-time variations in amplitude and arrival time as a function of pore fluid pressure. These dynamic changes result from pore fluid buildup, diffusion and the re-equilibration of pore pressure throughout the sample.
